# Hospital Meetings, &C.

**Published:** 1898-07-16

**Authors:** 


### HOSPITAL MEETINGS, &C.

### INVALID CHILDREN'S AID ASSOCIATION.

The Right Hon. J. G. Talbot, M.P., presided at a meeting on Tuesday, 5th instant, held at Cambridge House, Camberwell Road, on behalf of the Invalid Children's Aid Association, and, in introducing the subject, pointed out that its title indicated the objects of the association—the supervision and the assistance of the invalid and crippled children of the London poor. There is no branch of the association in existence in the districts of Camberwell and Dulwich, and the idea of the meeting was to enlist sympathy and co-operation in the neighbourhood. The method of the association is to organise a service of local visitors who shall each take charge of and regularly look after one or more children, make friends with them, and, in a kindly and sensible way, help their parents and relatives to give them the assistance they require to alleviate their suffering, and, if possible, put them and keep them on the road to recovery and to self-support. The Rev. Faulkner Baily urged that, as citizens, the need should be felt for individual and organised effort in this direction. In his work there at Cambridge House he was constantly realising the necessity for the help of such an association, the characteristic of whose work was that it did not leave a child till the child wanted its assistance no longer. Dr. Warrington Haward, speaking as a hospital surgeon, said he had constant opportunities of gauging the extreme value of the association's services. The number of cases of various joint diseases—spinal, hip, and so forth—among the London poor was almost incredible; while rickets and infantile paralysis were terribly common. In very many of these cases the ministrations of the visitors of the association meant just the difference between temporary and permanent loss of power or deformity. The most depressing part of a hospital surgeon's work was the knowledge that though it was comparatively easy to prescribe, he was often sure his instructions would not be carried out, and that many partially-cured cases would come back as bad or worse than before. This association's mission was to prevent the children becoming hopeless burdens, leading lives of grief, and help them to become happy and self-supporting. Miss Hughes followed with observations from the nurses' point of view, and emphasised the benefit both to child and friends of the regular visits of a sensible, sympathetic, kindly, and tactful person, who can advise the mother as to the benefits of ventilation and cleanliness, and the necessity for steady and continuous attention. Colonel Dalbiac, M.P., and Canon Daniel also spoke and were followed by Miss Forestier Walker, who made a most telling and magnetic appeal from the point of view of the visitor. The resolutions pledging the meeting to extend the work of the association in the neighbourhood, and recording its opinion that the most helpful means of benefiting the invalid children of the poor was by the watchful care of local visitors pledged to help them, were carried unanimously.

### WEST HAM HOSPITAL.

The seventh annual festival dinner of the West Ham Hospital was held under the presidency of Mr. A. Boake at the Great Eastern Railway Hotel on Thursday, the 7th inst. After the usual loyal and other toasts, the Chairman proposed "Prosperity to the Hospital," which, he said, was situated in the midst of a teeming working-class population, the hospital needs of which were very great. The borough of West Ham had about 275,000 inhabitants, the great majority of whom were artisans, and for this population they had one small hospital with sixty beds, forty of which only were available, as twenty had to be reserved for any great casualty which might arise in the great factories which surrounded the hospital or for such disasters as had occurred lately at the launching of the battleship *Albion*. He contrasted this state of affairs with the situation in Dublin, his native city, where in the metropolitan area there were about 250,000 inhabitants, and eighteen hospitals possessing an average of about eighty-four beds per hospital. It was patent, he thought, that the West Ham Hospital ought to be supported nobly and generously, so that it should not only be maintained in its present state of efficiency, but that its operations might be largely extended. (Cheers.) The Secretary announced contributions amounting to about £460. Various other toasts were given.

### ST. PANCRAS INFIRMARY.

At the invitation of the Visiting Committee, on Friday, July 1st, a large number of friends went over the buildings and grounds of the St. Pancras Infirmary on the occasion of the annual inspection of the establishment. Fortunately the day was a fine one, though towards evening the rain threatened somewhat, and the visitors were enabled to enjoy the pretty grounds, enlivened by a band and the now fashionable Highland pipers, as well as to inspect the wards and the various offices. In the evening an entertainment was given to the patients, who plainly showed their appreciation of the various efforts to relieve their tedium and pain.

